# FBXW2 inhibits the progression of gastric cancer via promoting β-catenin ubiquitylation

**DOI:** 10.7150/ijms.108501

**Published:** 2025-03-24

**Authors:** Yanshen Kuang, Mu Ke, Weizheng Liu, Fengming Xu

**Affiliations:** Department of General Surgery, the First Medical Center, Chinese PLA General Hospital, Haidian District, No.28, Fuxing Road, Beijing 100853, China.

**Keywords:** FBXW2, β-catenin, GSK3β, gastric cancer, ubiquitylation

## Abstract

**Background:** F-box and WD-repeat-containing protein 2 (FBXW2), an E3 ubiquitin ligase, may play a crucial role in tumorigenesis. However, its function in gastric cancer remains unknown.

**Methods:** The expression levels of FBXW2 and β-catenin in gastric cancer samples were analyzed using RT-PCR and immunohistochemistry, with Pearson correlation analysis to assess their relationship. AGS and HGC-27 gastric cancer cells were transfected with sh-FBXW2, and their viability was evaluated using the CCK8 assay, while invasion ability was assessed via the transwell assay. Western blotting was performed to measure the expression levels of FBXW2, β-catenin, GSK3β, and Axin2 in AGS cells. Additionally, a ubiquitination assay was conducted to examine the effect of sh-FBXW2 on β-catenin ubiquitination. Immunoprecipitation was used to determine the potential interaction between FBXW2 and β-catenin.

**Results:** FBXW2 expression was downregulated, whereas β-catenin expression was upregulated in gastric cancer tissues compared to adjacent normal tissues, showing a significant negative correlation (*r* = -0.52, *P* < 0.001). Knockdown of FBXW2 (sh-FBXW2) promoted gastric cancer cell viability and invasion while increasing β-catenin expression and reducing GSK3β and Axin2 levels. Furthermore, FBXW2 was found to bind β-catenin and facilitate its ubiquitination, leading to enhanced nuclear translocation of β-catenin.

**Conclusions:** FBXW2 suppresses gastric cancer progression by promoting β-catenin ubiquitination, highlighting its potential as a therapeutic target.

## Introduction

Gastric cancer is the fifth most frequently diagnosed cancer and the third leading cause of cancer-related deaths worldwide 1-3. Due to its asymptomatic early stages, gastric cancer is often diagnosed at an advanced stage, leading to a poor prognosis. In patients with locally unresectable or metastatic disease, systemic and targeted therapies offer palliation, improved survival, and enhanced quality of life, although the median survival remains limited, ranging from 4 to 9 months 4, 5. Therefore, a deeper understanding of gastric cancer pathogenesis is crucial for developing novel therapeutic strategies.

Physiologically, the canonical Wnt-β-catenin pathway plays a crucial role in stem cell maintenance, differentiation, and proliferation 6-8. In gastric cancer, mutations leading to aberrant Wnt-β-catenin signaling activation occur in 30-50% of patients 9, promoting self-renewal and therapy resistance 10-12. These findings suggest that targeting Wnt-β-catenin signaling could present a promising therapeutic strategy for gastric cancer.

Various signaling factors regulate Wnt-β-catenin signaling through post-translational modifications. Among these, key molecules such as Axin, β-catenin, glycogen synthase kinase 3, and disheveled are primarily regulated by E3 ligase-mediated ubiquitination 13, 14. E3 ligases attach ubiquitins to target proteins, leading to their proteasome-mediated degradation. One of the 69 F-box proteins, F-box and WD-repeat-containing protein 2 (FBXW2), serves as a substrate recognition receptor for the SKP1-Cullin1-F-box complex 15, 16. Notably, FBXW2 has been identified as a tumor suppressor in multiple cancers 17-19. In lung cancer, FBXW2 ubiquitinates and degrades β-catenin, thereby inhibiting migration and invasion 20. Overall, FBXW2 functions as a tumor suppressor in several cancers by targeting key oncogenic proteins for degradation, thereby inhibiting tumor growth and metastasis. However, its expression patterns and prognostic significance vary among different cancer types. FBXW2 was more expressed in various cancers including stomach adenocarcinoma, prostate adenocarcinoma, liver hepatocellular carcinoma, kidney chromophobe, neck squamous cell carcinoma, colon adenocarcinoma and cholangiocarcinoma. Meanwhile, its lower expression was revealed in uterine corpus endometrial carcinoma, thyroid carcinoma, lung squamous cell carcinoma, lung adenocarcinoma, and kidney renal clear cell carcinoma 21.

However, the potential role of FBXW2-mediated ubiquitination in gastric cancer migration and invasion remains largely unexplored. In this study, we employ ubiquitination and immunoprecipitation assays to demonstrate that FBXW2 acts as a substrate recognition receptor for β-catenin, inhibiting the progression of gastric cancer.

## Methods & materials

### Tumor samples

Paired adjacent normal tissue and tumor tissue were collected from gastric cancer patients, who were not receiving chemotherapy or radiation therapy before tumor resection in the First Medical Center, Chinese PLA General Hospital (n=74). The diagnose was confirmed by a pathological examination based on the WHO classification and criteria 22. According to the median expression of FBXW2 in Real-time quantitative PCR assay, the patients was classified into low FBXW2 mRNA level group (n=37) and high FBXW2 mRNA level group (n=37). The clinicopathological information and consent for clinical research utilization were also obtained. The study was approved by the First Medical Center, Chinese PLA General Hospital, and written informed consent was derived from the participants.

### Cell proliferation and colony formation

AGS cells and HGC-27 cells obtained from American Type Culture Collection were cultured with Dulbecco's modified Eagle's medium (DMEM) at 37°C with 5% CO_2_. ShRNA FBXW2 hairpin sequence: 5'-CCGGGCCTTTGAAACCTCGTCATTACTCGAGTAATGACGAGGTTTCAAAGGCTTTTTG-3' was synthetized in Sangon Biotech Co. to target GCCTTTGAAACCTCGTCATTA sequence in FBXW2.

AGS cells, HGC-27 cells, shRNA FBXW2 transfected AGS cells or HGC-27 cells (2× 10^3^ cells/well) were incubated in 96-well plates for 24, 48, 72, and 96 hours. 10 μL CCK-8 reagent (HY-K0301, MCE) was added into each well, followed by a further 2-hour incubation. The optical density was measured with iMark Microplate Reader (Bio-Rad) at 450 nm.

Transfected or un-transfected AGS cells were cultured in six-well plate (1×10^3^ cells/well) for two weeks, which were further fixed with 4% paraformaldehyde. Crystal violet (Sigma) was utilized to stain the colonies, and the number of colonies was counted. Each experiment was conducted in triplicates.

### Transwell invasion assays

Transwell (8 μm, Corning) pre-coated with Matrigel (BD Biosciences) were put in 24-well plates. Transfected or un-transfected AGS cells (2 × 10^4^ cells/100 μL) incubated with serum-free DMEM medium were placed onto the upper side of the chambers. The lower chambers were cultured with 600 μL of DMEM medium. After 48 hours, cells migrated to the lower side of the chambers were stained with crystal violet (5%, Sigma) and counted in six random fields under the Nikon 80*i* microscope.

### Reverse transcription polymerase chain reaction

Total RNA isolated from normal adjacent and tumor tissues of gastric cancer patients with TRIzol Reagent (Thermo Fisher) was transcribed into cDNA with QuantiTect Reverse Transcription Kit (Qiagen). The amplification was detected with SYBR Green (Bio-Rad) on 7500 Real-Time PCR System (Thermo Fisher). *FBXW2* primer, forward 5'-GGACATGCCTGAACACACTC-3' and reverse 5'-CCAGGACTGTGCAAGAGAGA-3'; *GAPDH* primer, forward 5'-ACCCAGAAGACTGTGGATGG-3' and reverse 5'-TCAGCTCAGGGATGACCTTG-3'. *GAPDH* was used as internal control, and 2^-ΔΔCt^ method was utilized to quantify the data.

### Western blotting

Nuclear & Cytoplasmic Extraction Reagents (Thermo Fisher) was applied for cytoplasmic and nuclear protein extraction. BCA Protein Assay Kits (Beyotime) was used to detect the concentration of protein. The proteins were separated with 12% SDS-PAGE and transferred to PVDF membranes (Millipore), which were further incubated with primary antibodies (overnight, 4℃) and secondary antibodies (1 h, at room temperature). Primary antibodies against the following proteins: FBXW2 (PA5-18189, Thermo Fisher), GAPDH (PA1-988, Thermo Fisher), β-catenin (71-2700, Thermo Fisher), GSK3β (9315S; Cell Signaling Technology), Axin2 (PA5-25331, Thermo Fisher), β-actin (MA5-33078, Thermo Fisher), lamin B1 (PA5-19468, Thermo Fisher). Finally, ECL Western Blotting Kit (Thermo Fisher) was used for chemiluminescence detection on ChemiDoc XRS^+^ system (Bio-Rad).

### Ubiquitination assay

His-Ub and HA-FBXW2 co-transfected AGS cells were lysed with Signal-Seeker Ubiquitination Detection Kit (Cytoskeleton) and pull down with nickel-nitrilotriacetic acid (Ni-NTA) beads (Qiagen) for 4 h at room temperature. Anti-HA or anti-FLAG antibody was utilized to pull-down the relative complex, and subsequently detected with Western blotting assay using anti-β-catenin antibody (71-2700, Thermo Fisher) or anti-FLAG antibody (F7425, Millipore).

### Statistical analysis

Brown-Forsythe ANOVA followed by Dunn's multiple comparisons test or Mann Whitney test was utilized to indicate the significance of quantitative parameters between groups. Pearson analysis was used to indicate the correlation between with the relative intensities of FBXW2 and β-catenin in tumor tissues of gastric cancer patients. All statistical analyses were performed with GraphPad Prism.

## Results

### Down-regulated FBXW2 expression correlates with TNM stage and lymph node metastasis status

**Figure 1:**
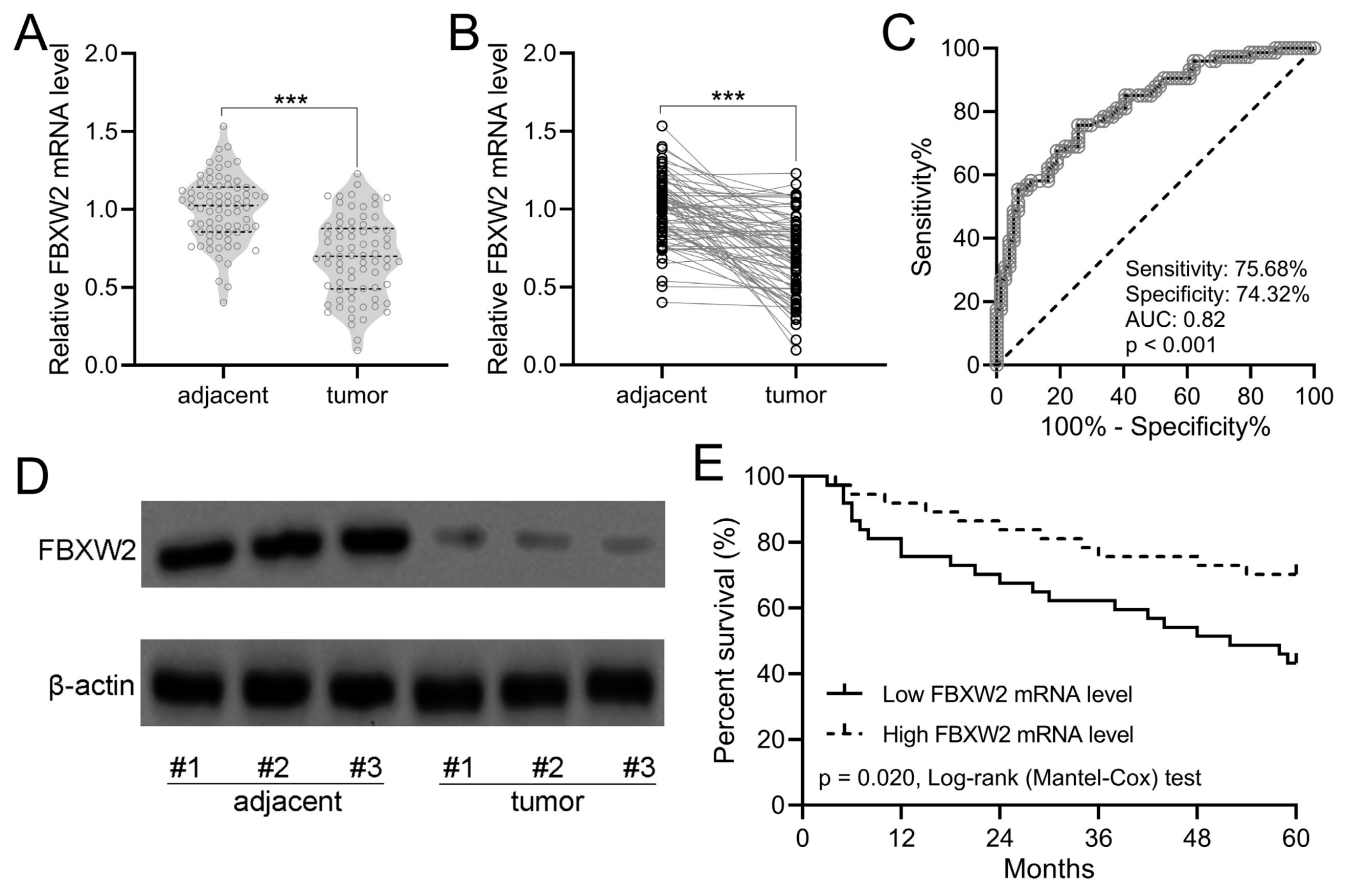
** FBXW2 was down-regulation in gastric cancer.** (A and B) FBXW2 expression in tumor and adjacent normal tissues of gastric cancer (GC) patients was assayed with RT-qPCR (n = 74). violin plot. *** *P* < 0.001 from *t*-test. (C) Receiver operating characteristic (ROC) analysis of FBXW2 expression in gastric cancer. (D) Representative Western blots of FBXW2 between adjacent normal and tumor tissues of gastric cancer patients. (E) Overall survival during 5 years in gastric cancer patients with the low or high expressions of FBXW2 mRNA levels according to the median of mRNA expressions normalized to adjacent tissues.

As shown in Fig. 1A with violin plot, the relative average FBXW2 expression was down-regulated significantly in human gastric cancer specimens compared with adjacent samples. In the withinstats plot (Fig. 1B), down-regulated FBXW2 expression was also revealed in gastric cancer specimens compared with paired adjacent samples. ROC analysis demonstrated that the relative expression of FBXW2 could be utilized to predict the occurrence of gastric cancer (sensitivity, 75.68%; specificity, 74.32%; AUC=0.82, *P*<0.001; Fig. 1C). On the other side, FBXW2 expression was correlated with TNM stage (*P*=0.034) and lymph node metastasis status (*P*=0.019) (Table 1). Down-regulated FBXW2 protein was observed in gastric cancer tissues compared with paired adjacent samples (Fig. 1D). It was further found that gastric cancer patients with high FBXW2 expression demonstrated significantly higher overall five-year survival rate than those with low FBXW2 expression (*P*=0.020, Fig. 1E). All of these indicated that down-regulated FBXW2 was correlated with the survival of gastric cancer.

### β-catenin demonstrates negatively correlation with FBXW2

**Figure 2:**
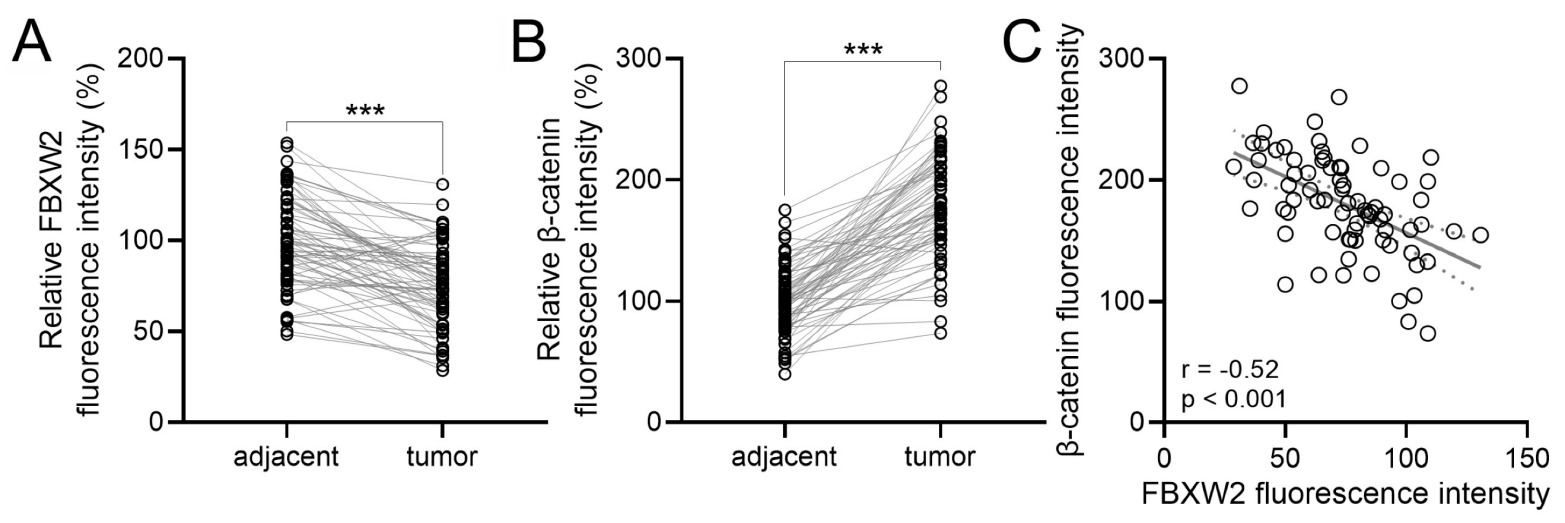
** The expressions correlation between FBXW2 and β-catenin in gastric cancer.** (A and B) The relative staining intensities of FBXW2 and β-catenin in adjacent normal and tumor tissues of gastric cancer patients. n = 74 for each group. Violin plot. *** *P* < 0.001 from paired *t*-test. (C) Pearson analysis of the relative staining intensities of β-catenin and FBXW2 in tumor tissues of gastric cancer patients. n = 74 for each group.

To evaluate the expression correlation of FBXW2 with β-catenin in gastric cancer, immunohistochemistry was performed in gastric cancer samples and the paired adjacent normal samples. Down-regulated FBXW2 expression (Fig. 2A) and up-regulated β-catenin expression (Fig. 2B) were detected in tumor samples compared with paired adjacent normal samples. A negative Pearson correlation was found between FBXW2 and β-catenin expression in gastric cancer patients (*r*=-0.52, *P*<0.001, Fig. 2C).

### FBXW2 inhibition promotes gastric cancer viability and invasion

**Figure 3:**
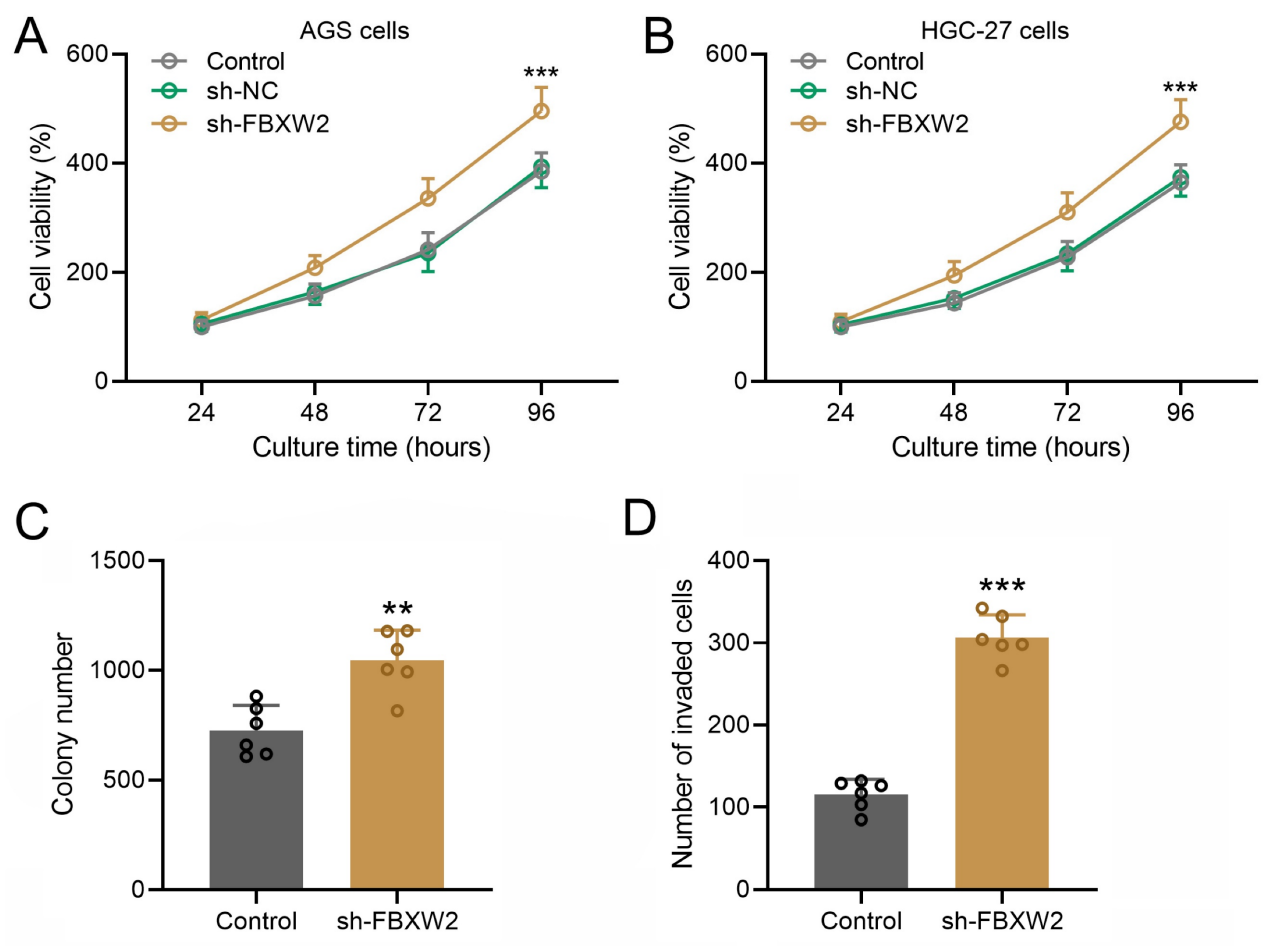
** Inhibition of FBXW2 promoted gastric cancer viability and invasion.** Sh-FBXW2 was transfected into AGS cells (A) or HGC-27 cells (B). Cell viability was measured by CCK8 after 24, 48, 72, and 96 hours after sh-FBXW2 transfection. ***P<0.001 compared to control. Brown-Forsythe ANOVA analysis was followed by Dunn's multiple comparisons test. (C) The colony formation assay of sh-FBXW2 transfected AGS cells was conducted 10 days after transfection and the number of colonies were calculated. (D) The invasion characteristics of sh-FBXW2 transfected AGS cells was observed by the transwell assay and invaded cells number was counted. Data were shown with mean ± SD. **P < 0.01, ***P < 0.001 compared to control. Mann Whitney test.

In both AGS (Fig. 3A) or HGC-27 cells (Fig. 3B), sh-FBXW2 transfection could promote the cell viability. The colony formation assay and Transwell assay were performed on sh-FBXW2 transfected AGS cells, and sh-FBXW2 transfection could promote the colony formation ability (Fig. 3C) and invasion ability of gastric cancer (Fig. 3D).

### FBXW2 inhibition alters the expression of Wnt/β-catenin signaling related molecules

**Figure 4:**
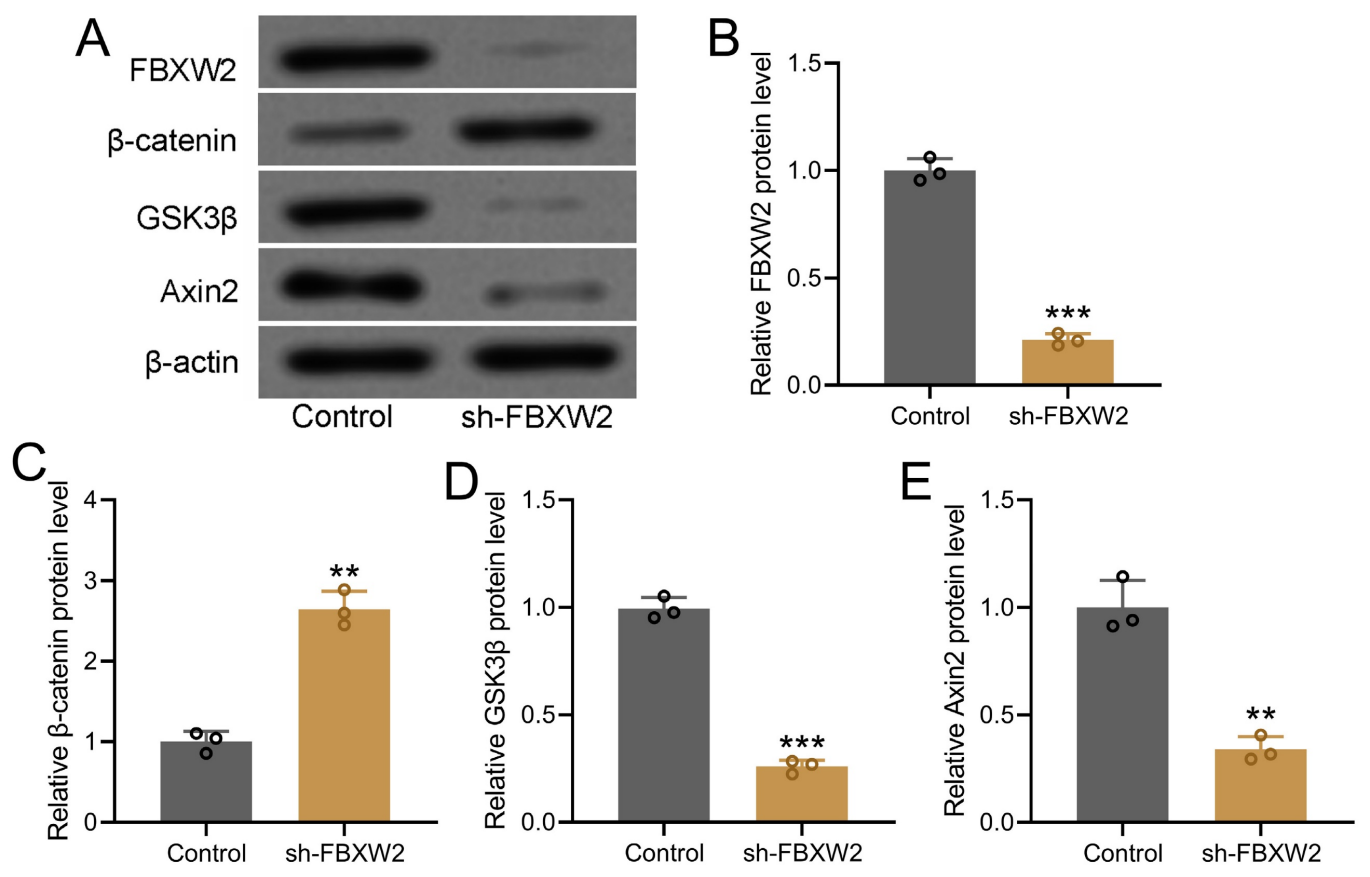
** Inhibition of FBXW2 activated Wnt/β-catenin signaling in AGS cells.** After sh-FBXW2 transfection for 72 hours, Western blotting was utilized to measure FBXW2, GSK3β, β-catenin, and Axin2 expression in AGS cells (A). The relative expression was normalized to control (B-E). Data were shown with mean ± SD. **P < 0.01, ***P < 0.001 compared to control. Mann Whitney test.

To further decipher the potential relation between FBXW2 and β-catenin, we screened the relative expression of WNT-β-catenin pathway related molecules in sh-FBXW2 transfected AGS cells by Western blot (Fig. 4A). Down-regulated FBXW2 was observed in sh-FBXW2 transfected AGS cells, which confirmed the success of sh-FBXW2 transfection (Fig. 4B). It was worth noting that up-regulated β-catenin (Fig. 4C), down-regulated GSK3β (Fig. 4D), and down-regulated Axin2 expression (Fig. 4E) was detected in sh-FBXW2 transfected AGS cells. All of these indicated that FBXW2 inhibition could activate the expression of Wnt/β-catenin signaling related molecules.

### FBXW2 can bind with β-catenin to accelerate the ubiquitination

**Figure 5:**
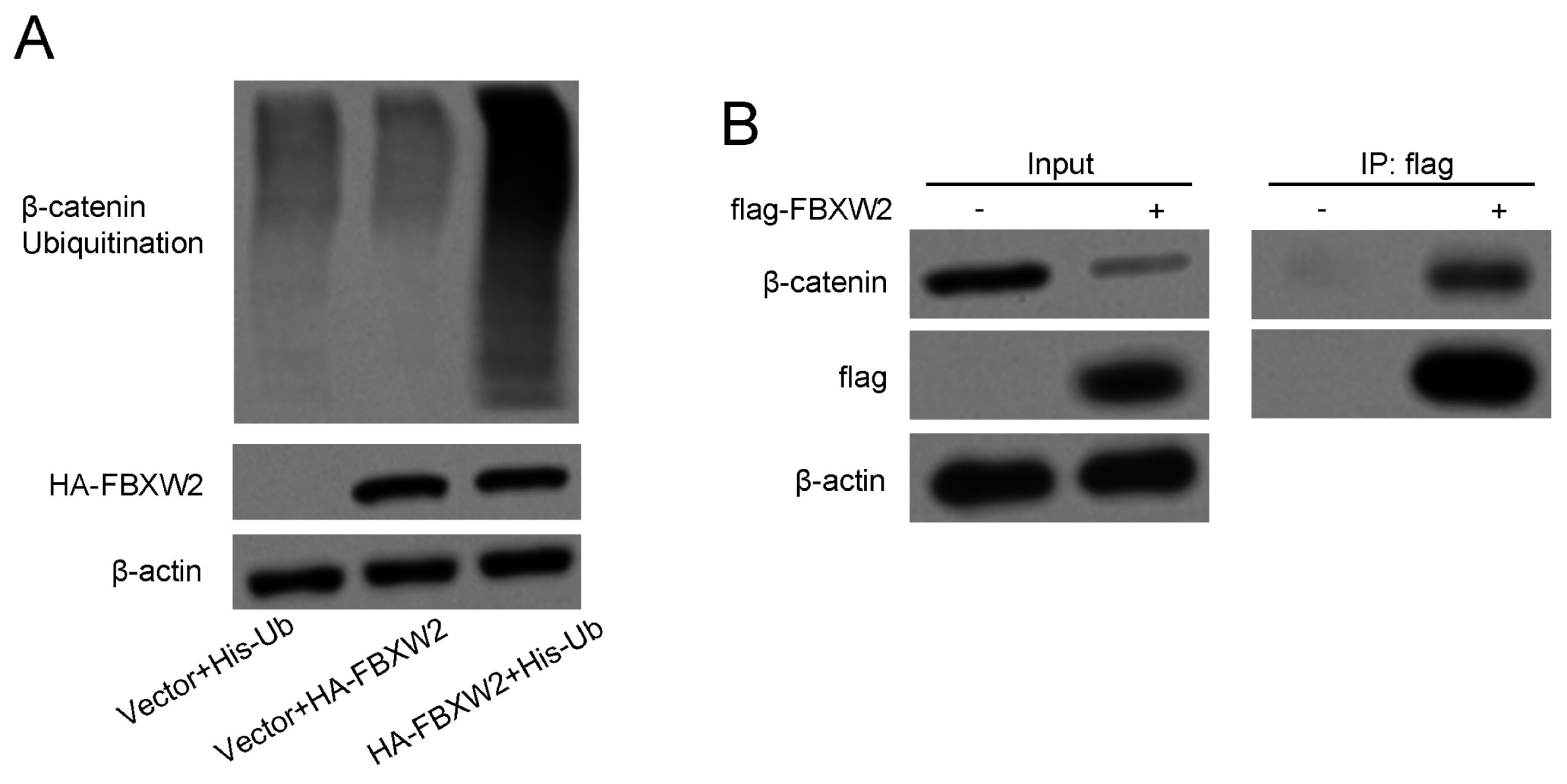
** FBXW2 can bind with β-catenin to accelerate the ubiquitination of β-catenin.** (A) Indicated plasmids transfected AGS cells were lysed and pull-down with nickel-nitrilotriacetic acid (Ni-NTA)-beads. Ubiquitination level of β-catenin was detected by Western blotting. (B) Binding of FBXW2 with β-catenin was determined by Western blotting.

Whether FBXW2 could function as a substrate recognition receptor to bind with β-catenin and induce further ubiquitylation reaction was revealed. FBXW2 inhibition could significantly diminish endogenous β-catenin ubiquitylation as demonstrated by the *in vitro* ubiquitylation assay (Fig. 5A). On the other hand, immunoprecipitation assay also confirmed that FBXW2 could bind with β-catenin (Fig. 5B), which demonstrated that FBXW2 could mediate β-catenin specific ubiquitylation.

### FBXW2 inhibition promotes β-catenin nuclear translocation

**Figure 6:**
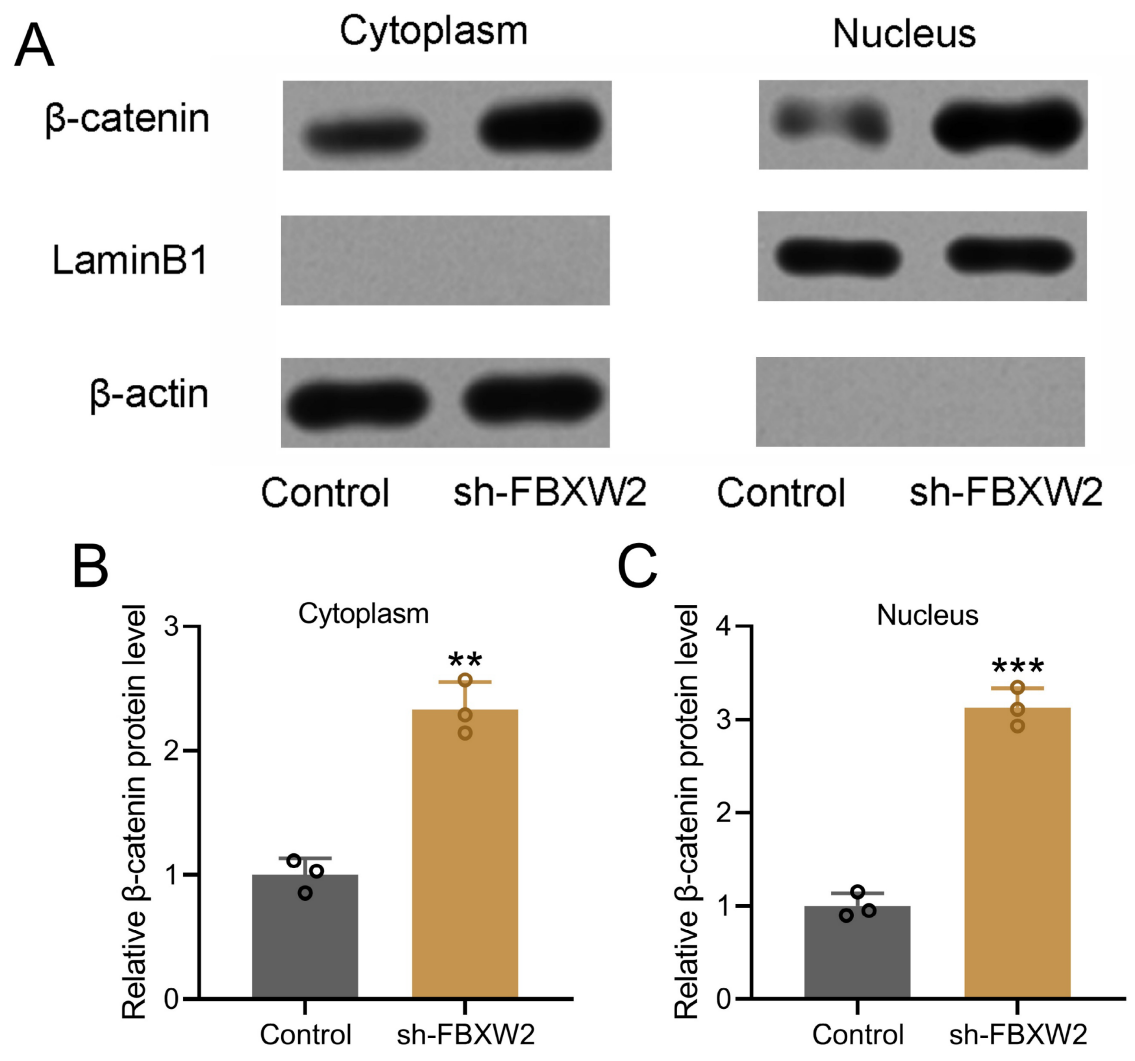
** FBXW2 inhibited β-catenin nuclear translocation.** (A) AGS cells were transfected with sh-FBXW2 for 72 hours. The expressions of β-catenin in cytoplasm and nucleus (A) were detected with Western blotting. β-actin and laminB1 were utilized as loading controls and β-catenin expression was normalized to control (B and C). Data were shown with mean ± SD. **p < 0.01, ***p < 0.001 compared to control. Mann Whitney test.

Nuclear and cytoplasmic protein extraction and Western blot were performed to detect the relative expression of β-catenin (Fig. 6A). Our results demonstrated that sh-FBXW2 could promote the relative cytoplasmic expression (Fig. 6B) and nuclear expression (Fig. 6C) of β-catenin.

## Discussion

Ubiquitination of cellular signaling proteins plays a crucial role in the tumorigenesis, progression, and prognosis of gastric cancer 23, 24. In this study, we demonstrate that the ubiquitin ligase FBXW2 regulates the stability of β-catenin, supported by the following evidence: 1) FBXW2 binds to β-catenin and promotes its ubiquitination; 2) FBXW2 knockdown via shRNA increases the expression of Wnt/β-catenin pathway-associated molecules; 3) FBXW2 knockdown enhances β-catenin nuclear translocation. Additionally, clinical samples reveal a negative correlation between FBXW2 and β-catenin expression, with FBXW2 inhibition promoting gastric cancer cell viability, colony formation, and invasion. Collectively, these findings suggest that FBXW2 suppresses gastric cancer progression by facilitating β-catenin ubiquitination.

As a ubiquitin ligase, FBXW2 was initially identified for its role in promoting the polyubiquitination and degradation of glial cell missing 1, thereby suppressing placental cell migration and invasion 20. In prostate cancer, FBXW2 facilitates epidermal growth factor receptor ubiquitination and degradation, thereby inhibiting EGF-induced growth and metastasis 25. Additionally, FBXW2 functions as a tumor suppressor by inhibiting lung cancer cell proliferation and metastasis through the ubiquitination and degradation of S-phase kinase-associated protein 2 and β-catenin 20. In this study, we further demonstrate that FBXW2 promotes β-catenin ubiquitination and degradation, thereby suppressing gastric cancer growth and invasion.

In lung cancer cells, endogenous FBXW2 has been shown to bind with both wild-type and constitutively active β-catenin mutants 20. In gastric cancer, mutations in exon 3 of β-catenin are frequently observed 26. However, the specific binding sequence of FBXW2 with β-catenin in gastric cancer remains unclear and requires further investigation. Both Wnt-dependent and Wnt-independent mechanisms can stabilize β-catenin nuclear translocation, allowing it to function as a transcriptional coactivator that promotes the expression of genes associated with proliferation and invasion. Understanding the interaction between FBXW2 and mutant β-catenin could provide valuable insights into gastric cancer progression and represents an intriguing avenue for future research.

Previous studies have demonstrated that low expression of FBXW7 contributes to gastric carcinogenesis and poor prognosis 27, a trend that aligns with our findings on FBXW2. In this study, we confirmed that FBXW2 plays a crucial role in regulating cell viability and invasion in gastric cancer. Notably, FBXW2 is prone to mutations, which may alter its biological function and potentially impact overall prognosis 28. Additionally, FBXW2 interacts with other ligand proteins to form the SKP1-Cullin1-F-box complex, mediating ubiquitination. Therefore, its clinical application requires careful evaluation to ensure therapeutic efficacy and safety.

## Conclusions

Overall, our study demonstrates that FBXW2 inhibits gastric cancer progression by promoting β-catenin ubiquitination, highlighting its potential as a therapeutic target.

## Figures and Tables

**Table 1:** The relationship between the expression level of FBXW2 and clinicopathological factors in gastric cancer patients (n = 74).

Variables	FBXW2 mRNA level		p value
	Low (n = 37)	High (n = 37)	
Gender			
Male	24 (64.9 %)	20 (54.1 %)	0.478
Female	13 (35.1 %)	17 (45.9 %)	
Age (years)			
< 60	15 (40.5 %)	18 (48.6 %)	0.640
≥ 60	22 (59.5 %)	19 (51.4 %)	
TNM stage			
I-II	16 (43.2 %)	26 (70.3 %)	0.034
III-IV	21 (56.8 %)	11 (29.7 %)	
Differentiation			
Poor	23 (62.2 %)	27 (72.9 %)	0.457
Well-moderate	14 (37.8 %)	10 (27.1 %)	
Lymph node metastasis			
Non-metastasis	15 (40.5 %)	26 (70.3 %)	0.019
Metastasis	22 (59.5 %)	11 (29.7 %)	

Fisher's exact test. TNM: Tumor Node Metastasis.
